# Navigating Alternative Medicine Preferences in Palliative Care: Challenges and Collaborative Solutions

**DOI:** 10.7759/cureus.77864

**Published:** 2025-01-23

**Authors:** Paula Cerqueira, Sara Pereira, Raquel Costa, Ana Garrido Gomes

**Affiliations:** 1 Internal Medicine-Medicina 2, Unidade Local de Saúde do Alto Minho, Hospital Conde de Bertiandos, Ponte de Lima, PRT

**Keywords:** alternative medicine, care plan, interdisciplinary collaboration, palliative care, team work

## Abstract

Integrating alternative medicine into palliative care presents unique opportunities and challenges for healthcare providers. This article examines two clinical cases that illustrate the complexities of navigating patient preferences for alternative therapies within evidence-based palliative care. The first case involves a 50-year-old woman with metastatic breast cancer who prioritized the use of herbal poultices and natural remedies following her faith as a Seventh-day Adventist. Despite her initial resistance to conventional treatments, the collaborative efforts of the palliative care team facilitated compromises that improved her quality of life, including the use of radiotherapy to manage bleeding and opioids for pain relief in her final days.

The second case highlights a 68-year-old man with advanced pulmonary fibrosis who preferred herbal infusions in his oxygen humidifier, homeopathic remedies, and Reiki while declining opioids due to concerns about side effects. The team addressed his symptom management needs through patient-centered communication and interdisciplinary collaboration, incorporating safer alternatives and supportive therapies without compromising his values. These cases underscore the importance of balancing respect for patient autonomy with the imperative to provide safe and effective care. Key strategies include interdisciplinary collaboration, empathetic communication, and incorporating complementary therapies that align with patient values while mitigating risks. By fostering trust and maintaining flexibility, palliative care teams can effectively address the diverse needs of patients who seek alternative medicine, ensuring holistic and patient-centered care. The discussion provides actionable recommendations for integrating alternative medicine preferences into palliative care practice, emphasizing the need for ongoing education, monitoring, and collaboration to navigate these complex situations effectively.

## Introduction

Palliative care is a holistic approach that prioritizes the quality of life for patients with severe, life-limiting illnesses [1]. It addresses physical symptoms and emotional, social, and spiritual concerns to provide care that aligns with patients’ values and preferences [2]. In this context, patient-centered care requires teams to respect individual choices while upholding clinical standards.

Many patients use alternative medicine to complement or replace conventional treatments in palliative care [3]. Cultural beliefs, dissatisfaction with traditional medical interventions, fear of side effects, or a desire to take a more active role in their care can motivate this preference [4]. While these therapies-ranging from herbal remedies and homeopathy to acupuncture and spiritual practices provide comfort or align with personal beliefs, they often pose challenges for healthcare providers [5]. Key concerns include safety risks, lack of robust evidence, and potential conflicts with evidence-based treatments [6]. Navigating these preferences involves striking a delicate balance for palliative care teams. On one hand, it is crucial to honor the patient’s autonomy and integrate their values into the care plan. Healthcare professionals have an ethical responsibility to ensure patient safety and provide treatments supported by scientific evidence. This dual obligation often creates complex ethical, clinical, and logistical dilemmas [7].

This paper explores two illustrative cases that highlight these challenges. The first involves a patient with metastatic breast cancer who relied on herbal poultices in alignment with her personal religious convictions and beliefs. The second case study involves a patient with advanced pulmonary fibrosis. This patient used herbal remedies, homeopathy, and Reiki while declining opioid use because of concerns about side effects and chemicals. Both cases underscore the importance of interdisciplinary collaboration, effective communication, and creative problem-solving in addressing the unique needs of patients who prioritize alternative medicine. The discussion provides insights into the implications of palliative care practice and offers detailed recommendations for improving care delivery in these situations.

## Case presentation

Case 1: natural bandages and breast cancer

The referring physician sent a 50-year-old woman with metastatic breast cancer, including widespread bone and liver metastases, to the palliative care team for symptom management and holistic support. The patient had her own religious convictions, which guided her firm belief in natural remedies. She expressed a preference for using herbal poultices and plant-based bandages to treat her condition. She explained that her faith and lifestyle focused on natural healing and avoiding pharmaceuticals. This preference for natural treatments posed substantial challenges for the palliative care team, especially as the patient declined conventional therapies, including pain medications such as analgesics. She was concerned about the synthetic nature of these drugs and the potential side effects. The team also had significant concerns regarding the risks of infection from using unsterilized herbal poultices on the ulcerated lesions caused by the metastatic disease.

As the disease progressed, the patient’s condition became more complex. One of the breast lesions bled persistently, resulting in severe anemia. This left her feeling extremely fatigued and drowsy, prompting her to reconsider her position on some medical interventions. After some discussion, the patient agreed to a short course of radiotherapy to control the bleeding, which reduced the blood loss and significantly improved her energy levels. Despite her initial resistance, she later accepted blood transfusions to address her anemia, which brought significant relief. Over time, as her disease continued to advance, her pain became increasingly unmanageable with non-pharmacological interventions alone. In her last days, the patient agreed to use prescribed medications, including opioids, to manage her pain, thus enhancing her comfort and overall quality of life. These decisions reflected a delicate balance between her deeply held spiritual beliefs and the need for effective symptom control.

To navigate the patient’s preferences while ensuring appropriate care, the palliative care team adopted a multifaceted, patient-centered approach. They first engaged the hospital’s chaplain, who was familiar with her personal religious convictions and beliefs, to gain a better understanding of the patient’s values and to foster trust. They consulted with a clinical pharmacist to assess the safety of the herbs the patient was using, checking for any potential risks or interactions with her existing treatments. The healthcare team integrated psychological support to help her manage the emotional and psychological challenges of her illness and treatment decisions. In parallel, the team placed a strong emphasis on infection prevention. They worked closely with the patient to educate her on safe wound care practices while respecting her autonomy and faith-based decisions. Following several discussions, the patient agreed to use sterile materials for her bandages and topical antibiotics to reduce the infection risk. The team also introduced complementary non-pharmacological interventions aligned with her beliefs, such as massage therapy and aromatherapy, to further support her pain management plan. By combining the patient’s preferred herbal remedies with modern medical techniques, the team ensured a holistic and effective approach that minimized risks while respecting her spiritual needs.

Case 2: herbs, homeopathy, and pulmonary fibrosis

Because of worsening dyspnea, a persistent cough, and a decline in his functional status, the palliative care team received a referral for a 68-year-old male patient with advanced pulmonary fibrosis. The patient expressed a strong preference for alternative treatments, including using herbal infusions in his oxygen humidifier, homeopathic remedies for symptom relief, and Reiki sessions for overall well-being. He was steadfast in his refusal of opioids, citing concerns about potential side effects, including sedation, and a strong aversion to “chemical” medications.

The patient’s reliance on herbal infusions raised concerns among the palliative care team, particularly regarding the potential risks of airway irritation or contamination within the oxygen delivery system. His refusal of opioids also left his severe dyspnea and associated anxiety inadequately managed, which posed significant challenges for the team. Despite these issues, the patient expressed satisfaction with his alternative therapies and was resistant to exploring conventional treatments. The palliative care team adopted a collaborative, patient-centered approach to navigate these challenges while respecting the patient’s preferences. The team’s clinical pharmacist explained the risks associated with using herbal infusions in the oxygen humidifier and suggested safer alternatives, such as using aromatherapy oils for respiratory support, which better aligned with the patient’s preferences. In addition, the team guided a discussion on homeopathic limitations, suggesting supplemental oxygen and low-dose benzodiazepines for anxiety and comfort. Nursing staff took on a crucial role in managing the safe use of the oxygen humidifier, providing respiratory therapy education, and careful monitoring for potential complications arising from the patient’s alternative healthcare methods. In addressing the patient’s emotional and spiritual needs, the team included Reiki therapy in his care plan, working with a certified practitioner who provided sessions alongside the healthcare team and the family. This integration offered the patient a sense of empowerment and alignment with his personal beliefs.

As the disease progressed, the patient agreed to use supplemental oxygen more regularly, which helped ease his dyspnea and improve his comfort. Despite the continued refusal of opioids, the patient eventually agreed to trial nebulized lidocaine for cough relief, which provided partial symptom control and aligned with his preference for non-pharmacological interventions. Through ongoing support and flexibility, the team ensured that the patient’s care remained effective, respectful of his values, and in line with his beliefs.

## Discussion

These cases underscore the complexity of integrating alternative medicine into palliative care. Key challenges include balancing respect for patient autonomy with the responsibility to provide evidence-based care, addressing potential safety risks, and navigating family dynamics [2].

In Case 1, the team’s effort to engage a chaplain and an integrative medicine specialist highlighted prizing interdisciplinary collaboration. The team compromised on wound care practices to reduce infection risk. This involved collaboration with nurses on sterile techniques, patient education, and respecting patient beliefs and preferences. However, the case also showed the limits of alternative medicine in addressing severe symptoms, emphasizing the need for ongoing patient education about realistic outcomes.

In Case 2, integrating Reiki and changing herbal therapies within a safety framework exemplified prizing tailoring care to individual preferences. The patient’s eventual acceptance of supplemental oxygen and nebulized lidocaine showed how open communication and trust-building can lead to incremental changes that improve quality of life without undermining autonomy.

The broader implications highlight several critical considerations for palliative care teams. First, we cannot overstate the role of empathetic communication [1]. Patients seeking alternative therapies often do so out of held beliefs, cultural influences, or previous negative experiences with conventional medicine [3]. Building trust and fostering open dialogue enables collaborative decision-making that respects the patient’s autonomy while addressing clinical concerns. Second, integrating alternative medicine into palliative care underscores the need for continued education among healthcare providers [4]. This includes understanding the potential risks and benefits of various alternative therapies, as well as being equipped to discuss evidence-based findings with patients in a nonjudgmental manner. Interdisciplinary collaboration is key. Providers should work with integrative medicine specialists, chaplains and other professionals to create safe and meaningful care plans for patients [3].

These cases emphasize the importance of flexibility and creativity in palliative care. While evidence-based medicine remains the cornerstone of practice, the ability to adapt and incorporate alternative therapies can enhance patient satisfaction and trust [4]. This approach requires a willingness to explore compromises, prioritize patient values, and reassess the care plan as the patient’s condition develops. Respect, open communication, and collaboration are key to integrating alternative medicine into palliative care. This approach improves the quality of care for patients with diverse needs and beliefs [1].

Strategies for integrating alternative medicine preferences

The field of integrating alternative medicine in palliative care remains controversial [8]. One significant area of debate is the lack of robust evidence supporting many alternative therapies, which contrasts with their widespread popularity among patients [8,9]. Critics argue that incorporating these practices may divert attention from evidence-based interventions, while proponents highlight their role in holistic and patient-centered care. The divergence between clinical evidence and patient preference creates ongoing tension [9].

In recent years, there have been notable achievements in understanding the role of alternative medicine in palliative care [10]. Studies published as recently as 2022 emphasize the growing evidence for integrating certain complementary therapies, such as mindfulness meditation and acupuncture, in managing symptoms like chronic pain, nausea and anxiety [11]. For instance, a 2019 systematic review highlighted the efficacy of acupuncture in reducing cancer-related fatigue and chemotherapy-induced neuropathy, providing a pathway for safer symptom management without a pharmacological burden [12]. We attribute the observed effects in these cases-such as the gradual acceptance of conventional therapies-to the use of trust-building strategies, collaborative care models and empathetic communication. The patients’ openness to interventions like radiotherapy or supplemental oxygen emerged from sustained dialogue, education and a focus on aligning medical recommendations with their values. These results suggest that patient resistance to conventional medicine is not static but can develop when providers engage with patients' beliefs [12].

It is also essential to use a multifaceted and detailed approach to address the challenges presented by patients in palliative care who seek alternative medicine [13]. Grasping a patient’s motivations, beliefs, and preferences necessitates a thoughtful, individualized approach that goes beyond routine assessments [14]. A patient-centered strategy also involves leveraging the unique skills of an interdisciplinary team. Pharmacists can assess drug-herb interactions and adjust medication regimens to optimize safety, while integrative medicine specialists can provide evidence-based guidance on alternative therapies [15]. In addition, spiritual care providers and social workers play a critical role in addressing emotional and existential concerns, fostering a holistic framework for care. This synergistic approach honors patients’ values and maintains a firm commitment to safe and effective clinical practices.

Also, fostering open and empathetic communication is crucial for building trust and ensuring shared decision-making [16]. Teams can use motivational interviewing techniques to explore patients’ preferences, clarify misconceptions, and educate them on their chosen therapies’ potential risks and benefits. This non-judgmental dialogue helps patients feel heard and valued, which can enhance their willingness to consider evidence-based recommendations. Monitoring is necessary to evaluate the integrated care plan's effectiveness and safety [15]. Regular follow-ups allow the team to assess the patient’s response to conventional and alternative therapies and make adjustments to optimize outcomes. Consistent documentation of these evaluations ensures accountability and facilitates informed decision-making. Providing education and training for palliative care teams is essential to equip them with the skills needed to navigate complex scenarios involving alternative medicine [16]. Training programs should emphasize cultural competence, ethical considerations, and the fundamentals of various alternative therapies. This knowledge enables clinicians to engage in informed discussions and address patients’ concerns.

The creation of institutional guidelines for managing alternative medicine requests can streamline decision-making and reduce ambiguity [17]. However, the high variability in alternative medicine practices, ranging from herbal remedies to energy-based therapies, poses a significant challenge to creating comprehensive and adaptable guidelines. To address this, institutions can establish flexible frameworks that focus on principles rather than rigid protocols. These frameworks might include standardized risk assessment tools, decision-making pathways that prioritize patient safety, and simple strategies for resolving disagreements within the care team or with family members. Interdisciplinary input and updated policies reflecting emerging evidence will better equip institutions to address the challenges of alternative medicine while upholding ethical care.

## Conclusions

Integrating alternative medicine into palliative care requires a careful blend of evidence-based practices, cultural sensitivity, and flexibility. Trust, patient preferences, and adaptability are key for palliative care teams. These qualities help improve patients’ quality of life and manage ethical and clinical complexities in diverse treatments.

These cases highlight the intricate balance between respecting patient autonomy and ensuring the delivery of safe, effective palliative care. Through open communication, interdisciplinary collaboration, and innovative problem-solving, healthcare teams can address the unique needs of patients who favor alternative medicine. Understanding and respecting patient values, educating patients about alternative therapies, and maintaining a patient-centered approach are crucial.
